# Similar trajectories of psychological states predict marital satisfaction

**DOI:** 10.1162/imag_a_00466

**Published:** 2025-02-18

**Authors:** Lei Li, Xinyue Huang, Qingyu Zheng, Jinming Xiao, Xiaolong Shan, Huafu Chen, Xujun Duan

**Affiliations:** The Clinical Hospital of Chengdu Brain Science Institute, School of Life Science and Technology, University of Electronic Science and Technology of China, Chengdu, PR China; MOE Key Lab for Neuro information, High-Field Magnetic Resonance Brain Imaging Key Laboratory of Sichuan Province, University of Electronic Science and Technology of China, Chengdu, PR China; School of Healthcare Technology, Chengdu Neusoft University, Chengdu, PR China

**Keywords:** fMRI, mate selection, marital satisfaction, inter-subject correlation, multi-voxel pattern analysis

## Abstract

Modern mate selection theories suggest that people are more likely to marrysomeone similar to themselves in terms of numerous attributes. Recent researchhas demonstrated a positive relationship between marital satisfaction andinter-subject correlation (ISC) of neural responses while viewing movies inmarried couples. Nevertheless, conventional ISC methods solely captureinformation about similarity in the temporal evolution of region-averaged neuralresponses, disregarding nuanced spatially distributed response topographies.Here, we integrated ISC and multi-voxel pattern (MVP) analysis to capitalizeinter-subject trajectory similarity (ISTS) of MVP. We demonstrated that marriedcouples showed significantly higher ISTS than randomly selected pairs, duringmovie viewing and resting state. The ISTS was particularly positively associatedwith marital satisfaction in married couples while viewing movies. In order toinvestigate latent “psychological states” characterized byrelatively stable patterns of MVP, a hidden Markov model was used to segment theneural events in married couples during viewing movies. We found the ISTS withinmanually defined events was a strong predictor of marital satisfaction. Theseresults suggest that married couples with high-level marital satisfaction mayexperience similar trajectories of mental states when exposed to a commonmarital-related stimulus, and extend our understanding of the neurobiologicalsignatures of intimate relationship.

## 1 Introduction

The proverb “Birds of a feather flock together” has long been a guidingprinciple in research on romantic attraction and marital satisfaction (Watson et al., 2004). Asubstantial body of literature consistently demonstrates that greater similaritybetween partners is associated with higher levels of marital satisfaction across avariety of attributes (Epstein &Guttman, 1984). These attributes can be broadly categorized into threedomains: demographic variables (e.g., age, religion, education, ethnicity, physicalattributes, attractiveness, intelligence quotient, and socioeconomic status),attitudinal domains (e.g., values, social attitudes, and interests), and personalitytraits (Gattis et al., 2004;Russell & Wells,1991). However, despite this overwhelming evidence, some studies haveintroduced nuances to the idea of similarity. For example, research has suggestedthat in certain cases, perfect similarity may not be ideal. Specifically, inindividuals who exhibit low levels of conscientiousness or extraversion, having apartner with higher levels of these traits may lead to greater marital satisfaction.This phenomenon, referred to as the “beneficial compensation effect,”suggests that dissimilarity in certain traits can complement and enhancerelationship dynamics (Shiota &Levenson, 2007). Due to these conflicting results, accumulating evidencesuggests that personality dimensions are not consistently associated with maritalsatisfaction (Rammstedt &Schupp, 2008;Watson et al.,2004). These studies primarily focus on comparing partners’similarity in characteristics measured through behavioral assays or self-reportmeasures. However, the similarity of one’s perceptions, thoughts, andfeelings about their surroundings are probably more important (Parkinson et al., 2018). Thus, itis plausible to examine the deeper similarities of couples, which reflects real-timemental responses.

Previous studies have reported that people tend to establish intimate relationshipswith others who reflect commonalities in perceiving, thinking, and reacting to theworld (Clore & Byrne,1974). Individuals’ interpretations and responses to their environmentincrease the predictability of their mutual thoughts and actions during socialinteractions (Berger, 1975).Moreover, when interacting with others who have similar thoughts, people willreinforce their own values, opinions, and interests and make positive affectiveresponses, thus promoting their attraction to each other (Parkinson et al., 2018). Previousstudies have demonstrated that the interpersonal synchronization provideshigh-quality communication (Jiang etal., 2012), and that high predictability allows more enjoyable socialinteraction (Zheng et al.,2018). Recent functional neuroimaging evidence suggests that the neuralactivities of two individuals are synchronized when they perform a cooperative or acompetitive task and that the level of inter-individual neural synchronization issignificantly associated with their similarity in perception and understanding oftheir surroundings (Cui et al.,2012;Stephens et al.,2010). In addition to complex cognitive tasks, functional neuroimagingdata acquired with the naturalistic paradigm provided valuable insights to detectmental processing (Wilson,2004). This method has several advantages over self-report measures incapturing individual differences in mental processing. It allows real-timeobservation of mental activities as they occur, eliminates the need for participantsto introspect about their mental states, and reduces self-presentation biases (King & Bruner, 2000).Furthermore, self-report questionnaires are typically constrained by a limitednumber of specific questions. In contrast, functional neuroimaging during the freeviewing of audiovisual stimuli allows for the simultaneous measurement of brainactivities associated with perceptual, cognitive, and affective processes. Numerousstudies have shown that inter-subject correlations (ISCs) of fMRI responses tonatural stimuli capture meaningful interpersonal similarities in how people attendto and make sense of what they see and hear. ISCs reflect differences in howparticipants, when instructed to adopt different mental perspectives, attend to,interpret, and store perceived information (Lahnakoski et al., 2014). Thus, revealing the temporalsequence of neural responses elicited by natural stimuli provides a meaningfulwindow into the unconstrained processing of these stimuli. This processing variesdepending on people’s personality traits, goals and values, and preexistingknowledge and assumptions (Berger& Calabrese, 1974). Therefore, intimate partners may beexceptionally similar in how they attend to, interpret, and emotionally react totheir surroundings. Indeed, recent findings suggest that social network proximitycorrelates positively with similarities in the temporal evolution of regional meanneural response amplitudes elicited by natural stimuli, especially in brain regionslinked to functions like attention allocation, narrative interpretation, andemotional responses (Parkinson et al.,2018). Modern mate selection probably makes more sense because similarityhelps marriage—after all, publicly blinding oneself to anotherperson’s loyalty and forward thinking, not only does a radical change inlifestyle for some, but of course, living day in, day out with the mate requires acertain degree of interaction. Recent studies have demonstrated that brainsimilarities that reflect real-time mental responses to subjective perceptions,thoughts, and feelings about interpersonal and social interactions are strongpredictors of marital satisfaction (Liet al., 2022), suggesting the association between inter-subjectsynchronization in time series of neural responses and marital satisfaction.However, extensive literature using multi-voxel pattern analysis (MVPA) on fMRI dataemphasizes the importance of examining not only univariate response magnitudes butalso spatially distributed response topographies (Haxby et al., 2011). Recent studies have integrated ISCand MVPA to investigate the similarity of the trajectory in neural response pattern(Chang et al., 2021;Nastase et al., 2019). If thetopology of the distributed responses of brain regions at a given moment can beconsidered as an approximate index of their mental state, then such an analysiscould provide insight into the evolution of a person’s mental state overtime. A recent study has shown that the temporal trajectories of MVPs in response tonaturalistic stimuli were unusually similar among friends and were associated tosocial networks (Hyon et al.,2020).

In the current study, we utilized individual differences in neural responses to testwhether couple-wise similarity in mental states, as indexed by temporal trajectoriesof MVPs, predicts marital satisfaction. We found that the inter-subject similarityof the trajectory in neural responses was positively correlated with maritalsatisfaction (highly satisfied married couples exhibited more similar trajectory ofpsychological state than less satisfied married couples). Additionally, previousstudies indicated that humans automatically segment continuous sensory input intodiscrete events (Baldassano et al.,2017). Therefore, by using modified Hidden Markov Model (HMM), weidentified event states in married couples across large-scale brain networks.Compared with high-level satisfaction married couples, the married couples withlow-level satisfaction represented fewer/longer events in dorsal attention anddefault mode networks during viewing movies. The similarity of the trajectory withinmanually event states was significantly correlated to the marital satisfaction.These findings demonstrate similar trajectories of mental states and provideadditional insights into the neurobiological basis of pair-bonding.

## 2 Materials and Methods

### 2.1 Ethics statement

This study was approved by the Institutional Review Board of the University ofElectronic Science and Technology of China and was conducted in accordance withthe Declaration of Helsinki. All the human participants provided writteninformed consent after the purpose and protocols of the study had been fullyexplained to them.

### 2.2 Participants

Forty-eight Chinese heterosexual couples, totaling 96 participants, wererecruited from local communities through flyers or internet advertisements. Allparticipants were right-handed and had an average age of 35.97 ± 6.1years. The couples had been married for at least 1 year (6.19 ± 5.25),and marriage was the first for both spouses. The initially published paperprovided a more detailed description of the data collection procedures (Li et al., 2022). In additionto the fMRI data from couples while they viewed marital and object-related movieclips, resting-state (eyes closed) fMRI data from this cohort of couples werealso included in this study. Here, we will describe the aspects of additionaldata collection and analysis that are most relevant to the new analysis.Following the Chinese Marital Quality Inventory (CMQI) scoring criteria, thecouples were categorized into two groups: those with high marital satisfaction(CMQI total scores >60) and those with low-level marital satisfaction(CMQI total scores <60). Marital satisfaction scores for each couple werecalculated by averaging the scores of both partners. In the fMRI data frommovie-viewing, 12 married couples were excluded due to high levels of headmotion. In the fMRI data from resting state, none couples were excluded.Therefore, resting-state fMRI data from all 48 couples and fMRI data from theremaining 36 couples while viewing naturalistic movies were used in furtheranalysis. Participants had no history of psychiatric or neurological disordersand were willing and eligible to participate in the fMRI study. All of them hadnormal or corrected-to-normal visual acuity. Written informed consent wasobtained from all the participants after fully explaining the purpose andprotocols of the study. This study was approved by the Institutional ReviewBoard of the University of Electronic Science and Technology of China and wasconducted in accordance with the Declaration of Helsinki.

### 2.3 Resting-state fMRI data acquisition

Participants were scanned using a 3T GE DISSOVERY MR750 scanner (GeneralElectric) with an eight-channel prototype quadrature birdcage head coil. Anecho-planar sequence (30-ms echo time; 2,000-ms repetition time; 3.75 ×3.75 × 3.2-mm resolution; 64 × 64 matrix size; flip angle =90°; 240 × 240-mm2 field of view; 43 interleaved transverse sliceswith no gap; 3.2-mm slice thickness) was used to acquire functional images. Theresting-state functional data consisted of 410 dynamic scans, with a totalfunctional data acquisition time of ∼13.6 minutes.

### 2.4 Preprocessing of resting-state fMRI data

Resting-state fMRI data were preprocessed using the Data Processing and Analysisof Brain Imaging toolbox (v4.3) (https://rfmri.org/dpabi). For each participant, the first 10 volumeswere discarded due to instability of the initial MRI signal. Slice-timingcorrection and head-motion realignment were performed on the remaining 400volumes, and all 48 couples were included with low levels of head motion duringscanning (i.e., head motion of either the husband or wife <3.0-mmtranslation or 3° rotation). Brain images from these participants werethen spatially normalized to a standard template for the Montreal NeurologicalInstitute (MNI) and resampled to 3 × 3 × 3 mm^3^. Thenormalized images were then linearly detrended to reduce the effects of signaldrifts. Nuisance covariates (24 parameters of head motion, white matter signal,cerebrospinal fluid signal, and global signal) were regressed out from the data(Friston et al., 1996).All the images were smoothed with a 6 × 6 × 6-mm^3^full-width at half-maximum Gaussian kernel. Linear detrending and bandpassfiltering (0.01–0.1 Hz) were subsequently performed to reduce the effectsof low-frequency drift and high-frequency noise. Data scrubbing was used toeliminate potential motion artifacts (Power et al., 2012). Signal outliers whose framewisedisplacement (FD) >0.5 mm with prior 1 and later 2 volumes were fitted tothe clean portion of the time series by using a third-order spline.

### 2.5 Region-of-interest identification

In the present study, we used the previously published whole-brain template(resampled to MNI152NLin2009cAsym standard space) with 200 brain regions (Hyon et al., 2020), each ofwhich was associated with one of the following brain networks fromYeo et al. (2011):seven-network parcellation—the visual (VN), somatomotor (SMN), dorsalattention network (DAN), ventral attention (VAN), limbic (LN), frontoparietaltask control (CEN), and default model network (DMN) (Yeo et al., 2011).

### 2.6 Inter-subject similarity in the temporal trajectory of MVPs

**Fig. 1.**
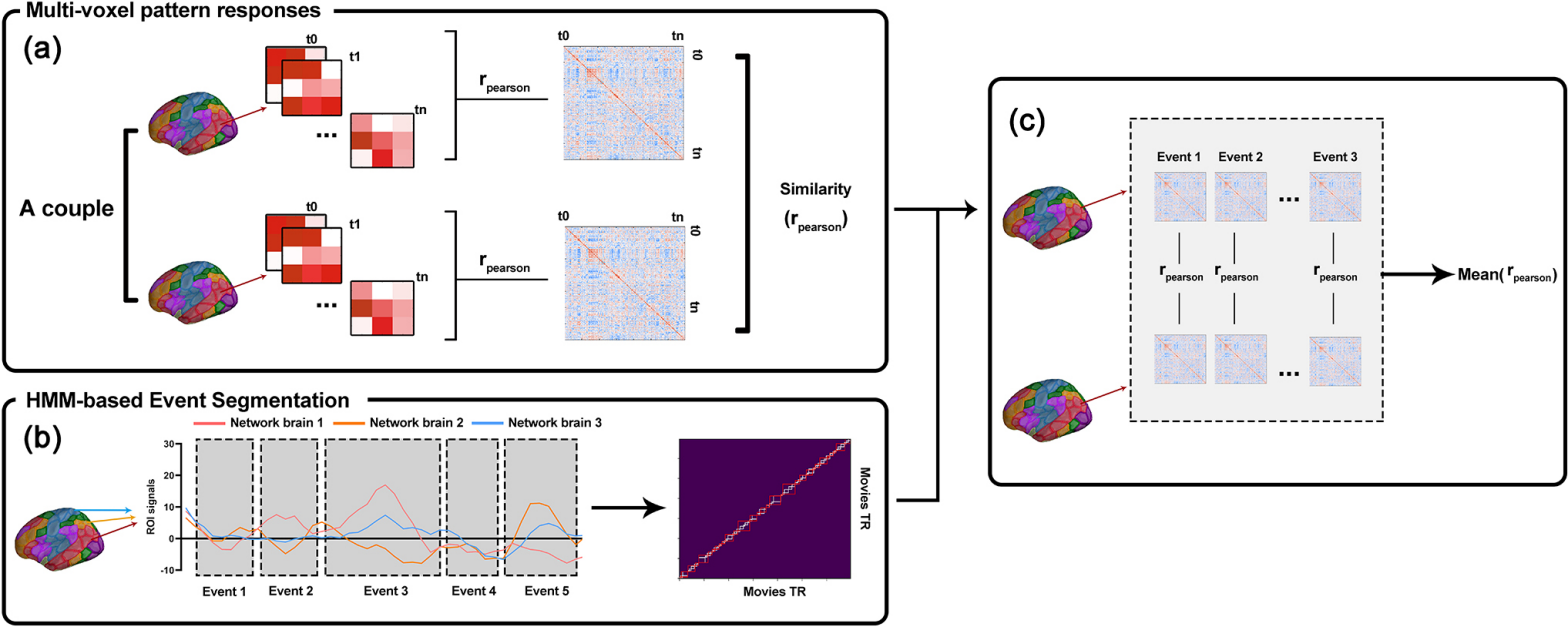
The analysis pipeline of inter-subject similarity in temporaltrajectories (ISTS). (a) For each ROI, the MVP characterized by thevectorized neural response of all the voxels was extracted at each timepoint for each subject. Pairwise correlations between MVPs across timepoints were then calculated, resulting in a TR x TR matrix, known as the“pattern trajectory matrix.” Each element of the patterntrajectory matrix reflects the degree of correlation between MVPs acrosstwo time points. The ISTS was obtained by calculating Pearsoncorrelations between the pattern trajectory matrices of couples. (b)Hidden Markov model was used to estimate two related components ofstable neural events in each of the large-scale brain networks:multivariate event patterns and their event structure (i.e., placementof boundaries between events). (c) Within each manually identifiedevents, pairwise correlations between MVPs across time points werecalculated to generate a pattern trajectory matrix. Inter-subjectcorrelations in corresponding event patterns for each dyad were thencalculated. These similarities were then averaged across events to yielda single mean correlation value characterizing within-events ISTS for agiven dyad for each ROI.

For both the resting-state and move-viewing fMRI data, in each of the brainregions, the MVPs were extracted for each time point, and the following analyseswere performed independently for each of the 200 each region of interests (ROIs)(Fig. 1a). We calculatedpairwise Pearson correlations between MVPs at each time point to construct amatrix that captured the trajectory of the MVPs for each participant (Chang et al., 2021;Nastase et al., 2019). In thismatrix, elements closer to the main diagonal represent pairwise correlationsbetween MVPs occurring closer in time, while elements further from the maindiagonal represent correlations between MVPs that are more temporally separated.In each ROI, Inter-subject trajectory similarity (ISTS) was defined as thePearson correlation between the vectorized trajectory structures of the husbandand wife, or between randomly selected male and female participants. Randommale-female pairings are generated by pairing a male with a randomly selectedfemale who is not married to the selected male, without repetition. Toillustrate how relative similarities in temporal trajectory in each brain regionvaried as a function of marital status, the ISTS was normalized (i.e., z-scored)across high-level marital satisfaction, low-level marital satisfaction, andrandom pairs for each region. The resulting similarity vectors (ISTS-Z scores)for each of the 200 anatomical ROIs were normalized to have a mean of zero and astandard deviation of one (Parkinson et al., 2018).

### 2.7 Relationships between inter-subject similarity in temporal trajectory and marital satisfaction

Whole-brain ISTS measure between married couples was obtained by averaging theISTS across all 200 ROIs. A 2 × 2 ANOVA with whole-brain ISTS as thedependent variable was used to determine the effects of fMRI state(movies-viewing vs. resting-state), marital status (high-level maritalsatisfaction vs. low-level marital satisfaction vs. random pairs), and theirinteraction. Between-group differences (high-level marital satisfaction vs.low-level marital satisfaction vs. random pairs) in the whole-brain ISTS whileviewing movies and in the resting state were assessed by using a two-sample*t*test (two-tailed). Pearson correlation was used todetermine the relationship between marital satisfaction and whole-braintrajectory similarity while participants were viewing movies and in the restingstate. Two-sample*t*tests were further performed on thewhole-brain ISTS while viewing marital and nonmarital movies between marriedcouples with high-level marital satisfaction and low-level marital satisfactionand random pairs. The significance threshold was set at FDR-corrected*P*< 0.05 for multiple comparisons.

### 2.8 Relation between region-wise inter-subject trajectory similarity and marital satisfaction

Partial least squares (PLS) regression was used to determine the relationshipbetween marital satisfaction and the regional ISTS in each of the 200 brainregions between couples. The ISTS of all brain regions were used as predictorvariables of marital satisfaction in the PLS regression (Abdi, 2010). The PLS-1 was thelinear combination of ISTS across regions that was the most strongly correlatedwith marital satisfaction. Permutation testing (5,000 times) was used to testthe null hypothesis that PLS-1 explained no more covariance between ISTS andmarital satisfaction than expected by chance. Bootstrapping (bootstrap samples= 500) was used to estimate the variability of each regionalISTS’s weight in PLS-1. The ratio of regional trajectory similarity tobootstrap SE was used to calculate z values. The confidence intervals (CIs) forthe ISTS in each brain region were calculated using z values. Brain regions thatreliably contributed to PLS-1 were subsequently identified after FDR correction(*P*< 0.05) for multiple comparisons.

### 2.9 Event segmentation by using Hidden Markov model (HMM) analyses

Previous research has shown that during realistic continuous perception,participants progress through a sequence of discrete event representations(hidden states) and that each event has a distinct (observable) signature (amulti-voxel fMRI pattern) that is present throughout the event (Baldassano et al., 2017).Notably, the optimal number and boundary of events varied across the cortex. Adata-driven event segmentation model based on an HMM was used and fitted to thefMRI data while the participants were viewing movies. This model temporallydivided the time into “events” with stable patterns of activitypunctuated by “event boundaries,” where the activity patternrapidly transitions to a new stable pattern. All HMM analyses were performedusing the Brainiak toolbox function,*Event Segmentation with HiddenMarkov Models*(Kumaret al., 2020). Each Hidden Markov Model (HMM) state was characterizedby a distinct mean activity pattern across all brain regions within thelarge-scale network parcellation. As indicated by prior studies, this analyticalframework incorporates the restriction that the HMM cannot return to a stateonce it has transitioned away from it. Put differently, each subsequent neuralpattern is assigned to either the same state as the preceding time step or to anew state that has not yet been visited (Baldassano et al., 2017). HMMs were trained on aversion of this Brainiak function that provides better fits when event statesare uneven in length (“split_merge=True”’ inBrainiak v0.10). To train the HMMs within each brain functional network, weinitially determined the optimal number of states using a nestedcross-validation approach. In each iteration of the outer loop of thisprocedure, a single test subject was selected, while the remaining 35 subjectswere divided into 27 for training and 8 for validation. The data from the 27training subjects were averaged, as were the data from the 8 validationsubjects. Within the inner loop of the cross-validation process, variousversions of the model were tested, ranging from 1 to 100 HMM states. For eachinner loop fold, the model was trained on the averaged data from the 27 trainingsubjects, applied to the averaged data from the 8 validation subjects, and thelog-likelihood of the fit to the validation set was computed. Based on theresults of the inner loop, we selected the number of states that maximized thelog-likelihood of the fit to the validation set. Subsequently, a new HMM wasfitted to the withheld “test” subject using this optimal number ofstates. After fitting the model to the fMRI data collected from couples whileviewing movies, we determined the optimal numbers and boundaries of events forseven large-scale brain networks (Fig. 1b). In order to investigate whether the perceived sensitivityto marital-related events is related to marital satisfaction, fitting modelswere estimated for two groups of married couples with high-level maritalsatisfaction and low-level marital satisfaction, respectively. We used thismethod to determine the optimal number of events for each functional network. Inthese analyses, the neural activity time courses of the different brain regionsin each network were used to determine the event boundaries of the activitypatterns of that network.

### 2.10 Relation between inter-subject trajectory similarity and marital satisfaction within manually defined events

For each subject, we extracted the MVPs at each time point. Event segmentationmodel results indicated that neural activity patterns in the DMN of marriedcouples with high- and low-level marital satisfaction have differentsensitivities to the perception of marital-related events. Therefore, temporaltrajectory matrixes of MVPs were then calculated across time points within eachof the corresponding defined events of the DMN. For each married couple, wecalculated Pearson correlations between subjects’ temporal trajectorymatrixes corresponding to the same events (Fig. 1c). Correlation coefficients were thenaveraged across events for each couple, resulting in a single within-event ISTSmeasure for each married couple for each brain region. In addition, theseanalyses were repeated for each of the movie-clips of fMRI stimuli to obtain thewithin-movie-clips ISTS. Between-group differences (high-level maritalsatisfaction vs. low-level marital satisfaction) of the whole-brain ISTS inmanually defined events and movie clips while viewing movies were assessed byusing a two-sample*t*test (two-tailed). PLS andbootstrapping-based significance tests, as described above, were performed toexplore whether and which of the within-events and within-movie-clips ISTS werepredictive of marital satisfaction.

## 3 Results

### 3.1 Inter-subject trajectory similarity of multi-voxel patterns and marital satisfaction

This study included 96 participants, comprising 48 heterosexual married couples,all of whom had been married for at least 1 year. The data reported here werealso used in our previously published study and were reanalyzed in this study.The initially published paper provided a more detailed description of the datacollection procedures (Li et al.,2022). In addition to the fMRI data from couples while they viewedmarital and object-related movie clips, resting-state (eyes closed) fMRI datafrom this cohort of couples were also included in this study. In the fMRI datafrom movie-viewing, 12 married couples were excluded due to high levels of headmotion. In the fMRI data from resting state, none couples were excluded.Therefore, resting-state fMRI data from all 48 couples and fMRI data from theremaining 36 couples while viewing naturalistic movies were used in furtheranalysis.

The originally published paper utilized an ISC approach, which focused onfluctuations in response amplitude and ignored the information contained indistributed spatial patterns of neural activity. In contrast, the present paperfocused specifically on spatially distributed response patterns (regardless oftheir overall magnitude) and, in particular, on individual differences in theevolution of such patterns over time during natural stimulation. For both theresting-state and move-viewing fMRI data, in each of the 200 ROIs, the MVPs wereextracted for each time point (Fig.1a). In each ROI, we calculated pairwise Pearson correlations betweenMVPs at each time point to construct a time-point-to-time-point matrix thatcaptured the trajectory of the MVPs over time in each subject (Chang et al., 2021;Nastase et al., 2019). Then,ISTS was defined as the Pearson correlation between the vectorized full-patterntrajectory structures of the husband and wife or between randomly selected maleand female participants. Random male–female pairs were generated bypairing each male with a randomly selected female (who was not married to him),ensuring no repetitions. To illustrate how the relative similarities in temporaltrajectory in each brain region varied as a function of marital status, the ISTSwas normalized (i.e., z scored across high-level marital satisfaction vs.low-level marital satisfaction vs. random pairs for each region). The resultingsimilarity vectors (ISTS-Z scores) for each of the 200 anatomical ROIs werenormalized to have a mean of zero and an SD of 1 (Parkinson et al., 2018).

**Fig. 2.**
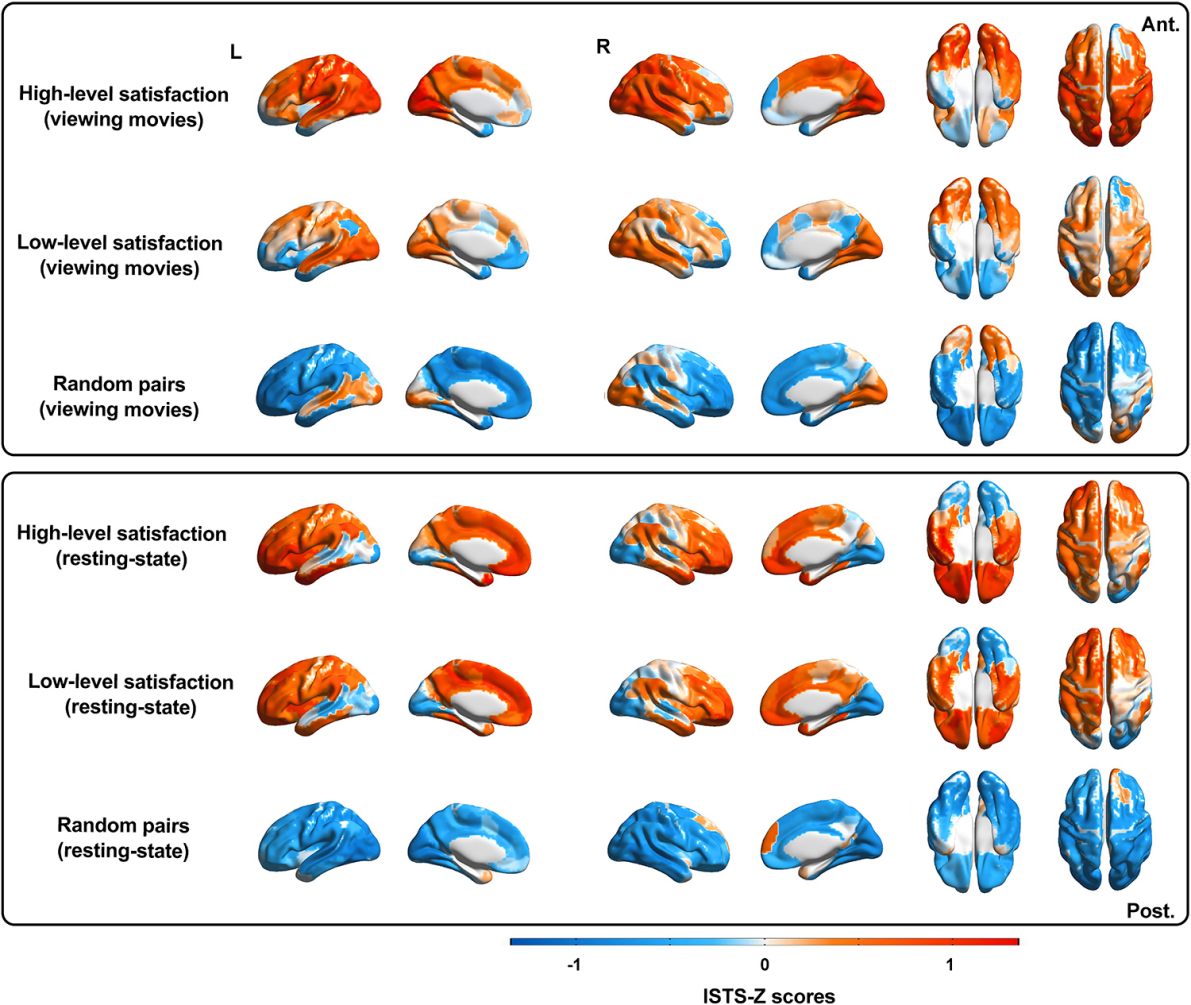
ISTS while viewing movies and resting state, averaged within levels ofmarital status. Married couples showed higher ISTS than random noncouplepairs at the whole-brain level while viewing movies and resting state.Married couples with high-level satisfaction showed higher ISTS thanmarried couples with low-level satisfaction during viewing movies. Toillustrate how relative similarities of overall ISTS varied as afunction of marital status, ISTS were normalized (i.e., z-scored acrossdyads for each region), averaged within marital status level, and thenprojected onto an inflated model of the cortical surface.

We first examined the relationship between marital satisfaction and trajectoriesof MVPs throughout the entire study. ANOVA revealed a main effect of maritalstatus (High-level satisfaction couples vs. low-level satisfaction couples vs.random pairs), with stronger ISTS-Z scores in married couples(*F*= 15.71,*P*< 0.001). Asignificant interaction effect was found between fMRI state and marital status(*F*= 14.23,*P*< 0.001).Compared with those of random pairs, the ISTS-Z scores were greater in marriedcouples while viewing movies (*t*= 57.42,*P*< 0.001, Cohen’s*d*= 2.37 [married couple with high-level satisfaction];*t*= 29.21,*P*< 0.001, Cohen’s*d*= 1.41 [married couple with low-levelsatisfaction]; FDR corrected) and in the resting state (*t*= 69.16,*P*< 0.001, Cohen’s*d*= 2.56 [married couple with high-levelsatisfaction];*t*= 60.41,*P*<0.001, Cohen’s*d*= 2.36 [married couple withlow-level satisfaction]; FDR corrected). Moreover, compared with couples withlow-level marital satisfaction, married couples with high-level maritalsatisfaction had higher ISTS-Z scores when viewing movies (*t*= 21.56,*P*< 0.001, Cohen’s*d*= 0.63, FDR-corrected) but not during restingstate (*t*= 1.05,*P*= 0.15,Cohen’s*d*= 0.13) (Fig. 2).

**Fig. 3.**
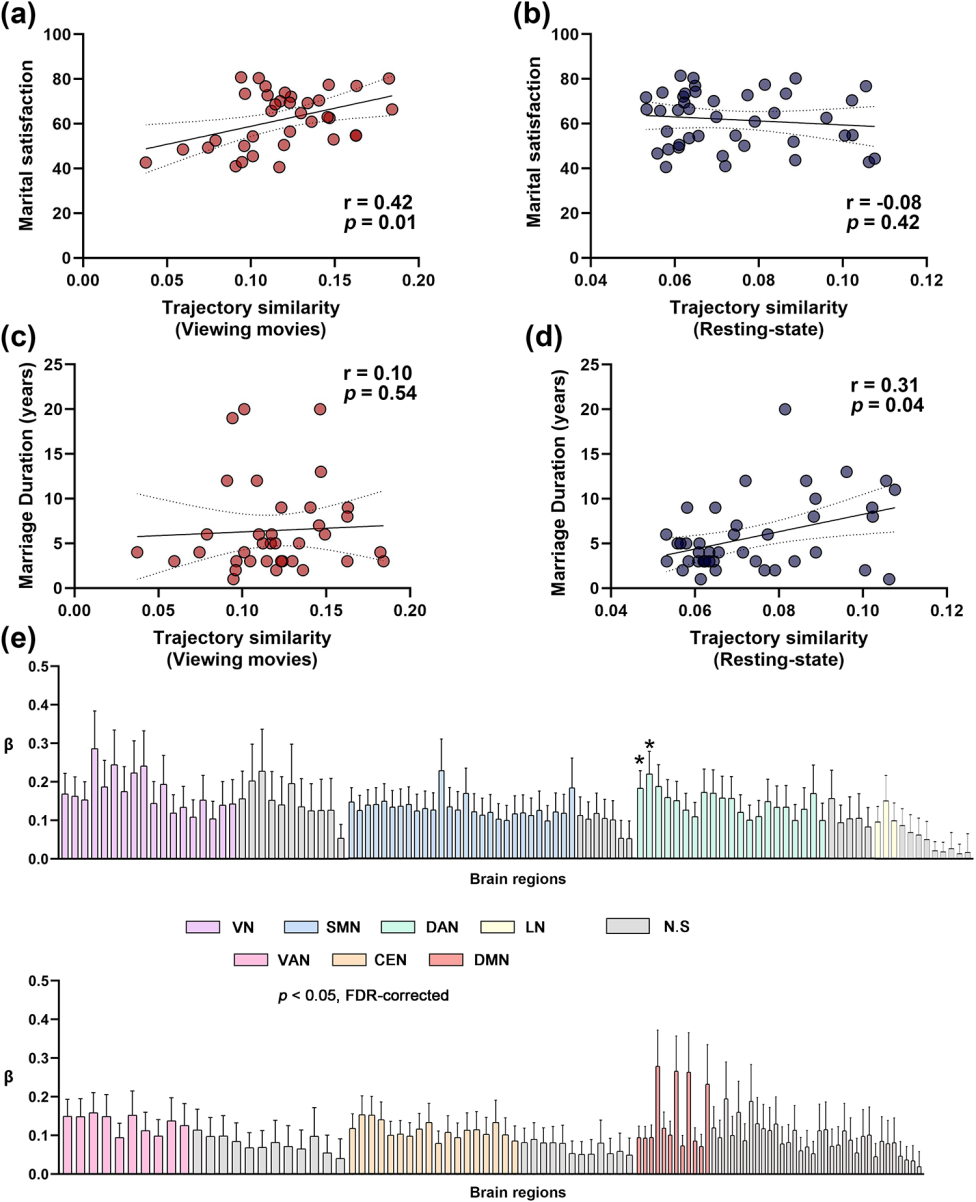
Relationships between ISTS across all time points and maritalsatisfaction for whole-brain and all brain regions. Correlation betweenaverage ISTS across the whole brain, and marital satisfaction whileviewing movie clips (a) and resting state (b). Correlation betweenaverage ISTS across the whole brain, and marriage duration while viewingmovie clips (c) and resting state (d). (e) PLS regression was conductedfor each brain region to predict marital satisfaction based on dyadicsimilarity in the trajectory of multi-voxel patterns across all timepoints. The regression coefficients for each brain region are presented,organized by their corresponding brain networks. Brain regions whereISTS significantly correlated with marital satisfaction are indicated byasterisks (*P*< 0.01, FDR-corrected,two-tailed).

Next, we examined whether the ISTS at the whole-brain level was related toindividual differences in marital satisfaction. We averaged the ISTS across all200 ROIs to obtain a whole-brain ISTS in married couples and investigated itsrelationship with marital satisfaction. We found that the whole-brain ISTS wassignificantly correlated with marital satisfaction while viewing movie clips(*r*= 0.42,*P*= 0.01) (Fig. 3a) but not during theresting state (*r*= -0.08,*P*=0.42) (Fig. 3b). These resultsdemonstrate that a higher-level inter-subject similarity in temporal trajectoryis associated with increased marital satisfaction while viewing movies inmarried couples. Additionally, we found that marriage duration during theresting state was significantly related to ISTS (Spearman correlation,*r*= 0.31,*P*= 0.01) (Fig. 3c), while there was nosuch correlation when viewing the movies (Spearman correlation,*r*= 0.10,*P*= 0.54) (Fig. 3d).

Next, we sought to identify the brain regions driving the relationship betweenthe ISTS and marital satisfaction. Partial Least Squares Regression (PLS)regression was used to determine the relationship between marital satisfactionand the ISTS in 200 ROIs during the viewing of movies. The first component ofthe PLS (PLS-1) was defined as the spatial map that captured the greatestfraction of the total ISTS (*P*_permutation_<0.001). After bootstrapping, FDR corrections were used to correct for multiplecomparisons across brain regions. We found that the ISTS in multiple brainregions contributed to marital satisfaction while viewing movies(*P*< 0.01; two-tailed, FDR-corrected). These regionswere involved in the DAN (Fig.3e). There were no brain regions in which the ISTS was significantlycorrelated with marital satisfaction in the resting state.

### 3.2 Inter-subject trajectory similarity of multi-voxel patterns while viewing marital and non-marital movies

**Fig. 4.**
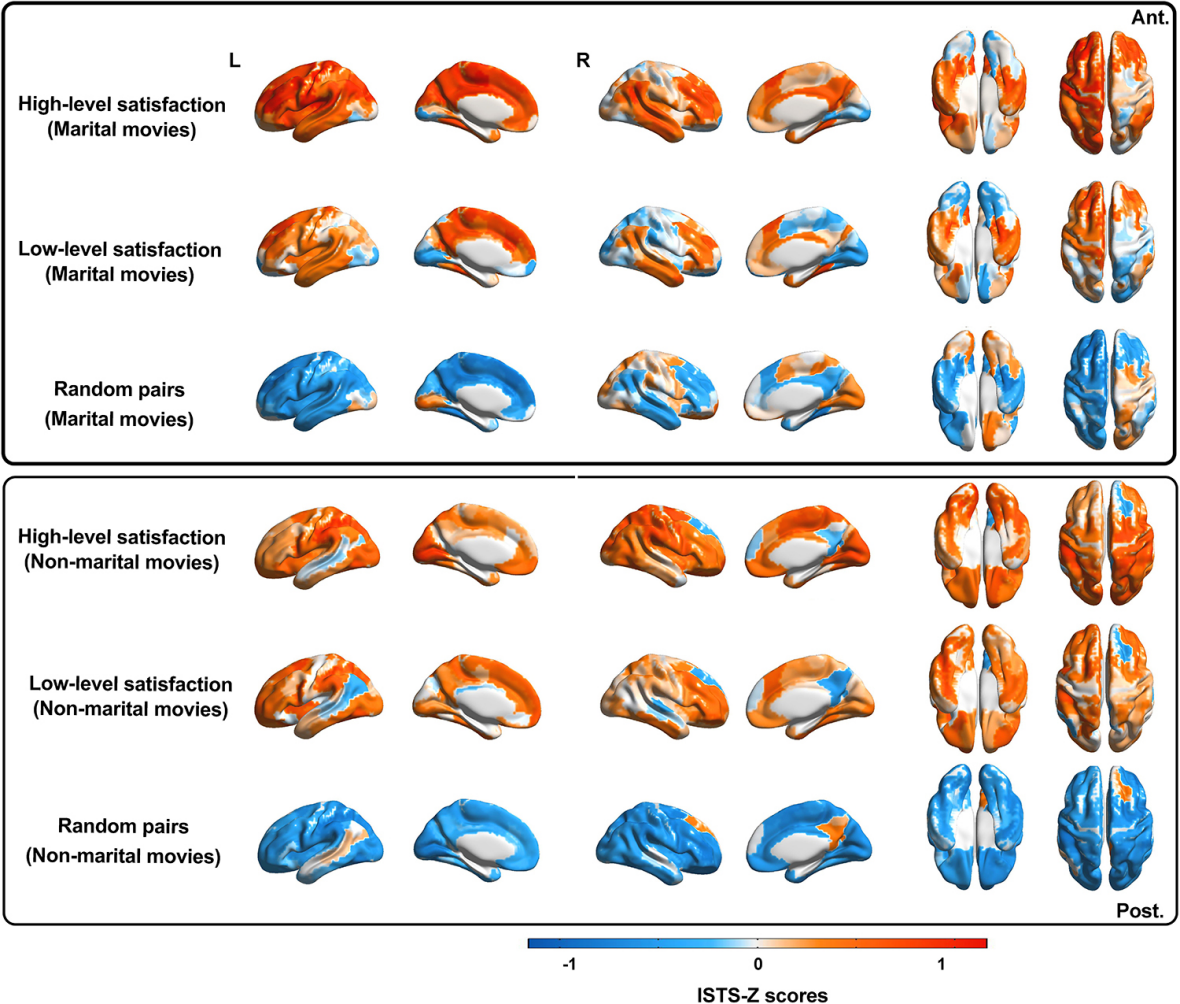
ISTS during viewing of marital and non-marital movie clips, averagedwithin levels of marital status. Married couples with high-levelsatisfaction showed higher ISTS than married couples with low-levelsatisfaction during viewing marital movies. The ISTS were alsonormalized (i.e., z-scored across dyads for each region) and averagedwithin marital status level.

During fMRI scanning, each participant was presented with two types of movieclips: six depicting scenes related to marital life, and six featuringnon-marital natural object-related scenes. Compared with that of random pairs,the ISTS-Z scores were higher in married couples when viewing marital movies(*t*= 21.16,*P*< 0.001,Cohen’s*d*= 2.07 [married couple with high-levelsatisfaction];*t*= 12.35,*P*<0.001, Cohen’s*d*= 1.25 [married couple withlow-level satisfaction]; FDR-corrected), and viewing non-marital movies(*t*= 34.82,*P*< 0.001,Cohen’s*d*= 3.26 [married couple with high-levelsatisfaction];*t*= 27.78,*P*<0.001, Cohen’s*d*= 2.61 [married couple withlow-level satisfaction]; FDR-corrected). Moreover, compared with couples withlow-level marital satisfaction, married couples with high-level maritalsatisfaction had higher ISTS-Z scores when viewing marital movies(*t*= 5.78,*P*< 0.001,Cohen’s*d*= 0.88, FDR-corrected) but not whenviewing non-marital movies (*t*= 1.46,*P*= 0.15, Cohen’s*d*= 0.38) (Fig. 4). These results indicatedthat married couples with high-level satisfaction showed higher levels ofbrain-wide neural trajectory similarity than those with low-level satisfaction.Furthermore, these findings demonstrated that this differentiation is specificto marital movies.

### 3.3 Inter-subject trajectory similarity in manually defined events

**Fig. 5.**
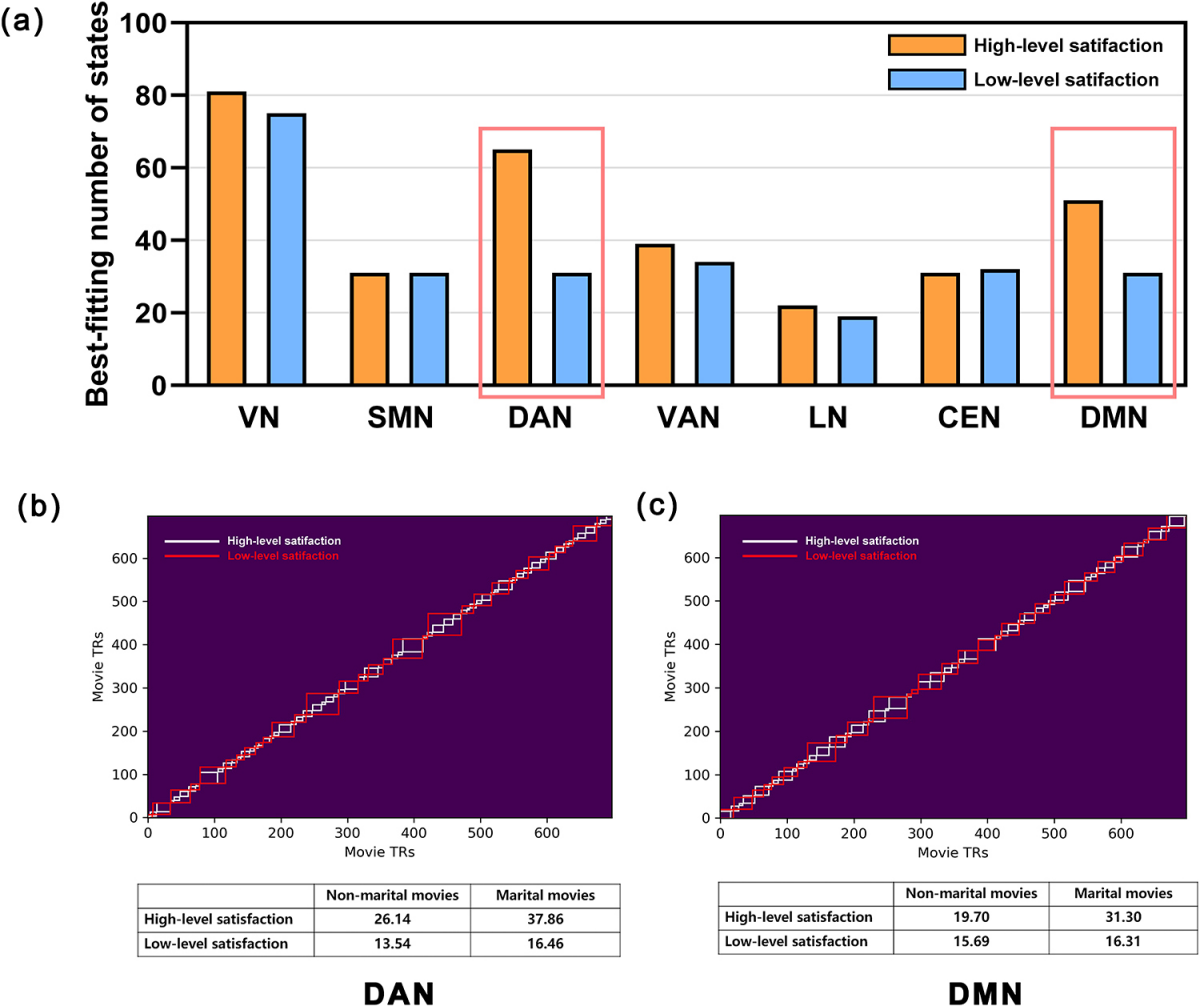
Event structure in married couples with high- and low-level maritalsatisfaction across large-scale brain networks. (a) The optimal numberof events for a given network was determined via a searchlight, whichfound the number of events that maximized the model log-likelihood inheld-out data. Compared with married couples with high-levelsatisfaction, the married couples with low-level satisfactionrepresented fewer/longer events in dorsal attention and default modenetworks during viewing movies. The model event boundaries found indorsal attention (b) and default mode networks (c) are outlined in white(married couples with high-level satisfaction) and red (married coupleswith low satisfaction).

Given that married couples process the movie in a similar way to each other, wenext investigated whether their neural activity contains evidence of eventsegmentation. We tested whether the perceived sensitivity to marital-relatedevents was related to marital satisfaction and captured signatures of thesegmentation of continuous experience in neural patterns during viewing ofmovies. A specialized HMM was employed to partition the network-level neuralresponse patterns into a reduced set of continuous, stable activity patternsthat align with event structures within the narrative (Baldassano et al., 2017). Thenumber of events that yielded the largest log-likelihood was identified as theoptimal number of events for each functional network. The neural patterns weredefined from all the regional ROIs in each functional network. We found that theoptimal number of events differed across the functional networks. Overall, wereplicated previous work showing that visual regions exhibit more events thanhigher-order associative regions (Yates et al., 2022). However, our results indicated that, comparedwith couples with low-level satisfaction, couples with high-level satisfactionhad more or shorter events in the DAN and DMN (Fig. 5a). Qualitative inspection revealed anotable association between the boundaries in the DAN and multiple types ofvisual changes in the movie, whereas boundaries in the DMN were specific tomarital-related cues. Specifically, compared to couples with low-level maritalsatisfaction, those with high-level marital satisfaction exhibited more eventsin the DAN, regardless of whether they were viewing scenes related to marital ornon-marital content. However, in regard to the DMN, couples with high-levelmarital satisfaction experienced more events only when viewing scenes related tomarriage (Fig.5b–c).

**Fig. 6.**
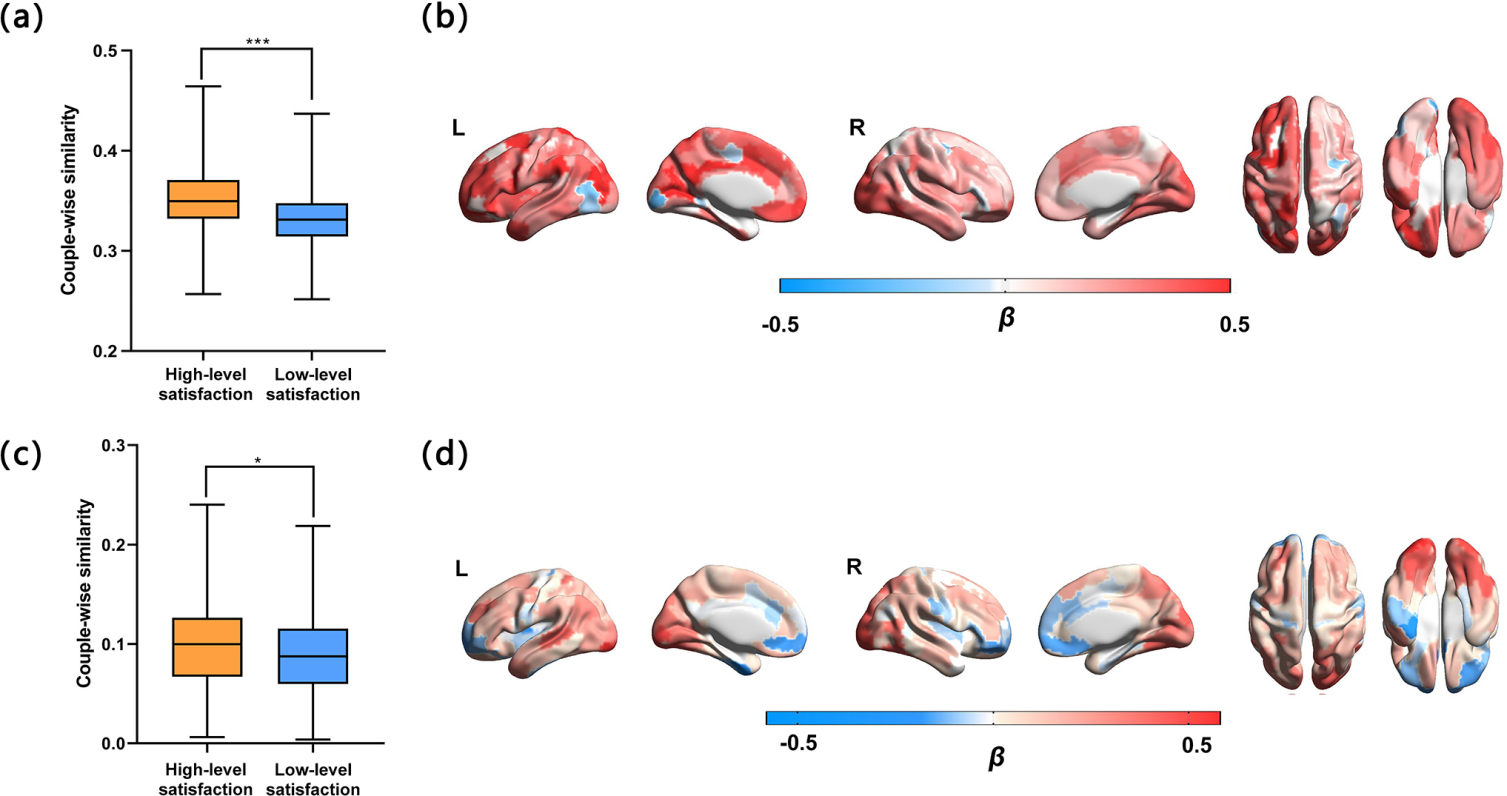
Marital satisfaction is correlated with whole-brain within-event ISTS inmarried couples. Group differences in average within-event ISTS (a) andwithin-movie-clips ISTS (c) across the whole brain during viewing ofmovies in couples with high and low-level marital satisfaction. Brainareas in which within-event ISTS (b) and within-movie-clips ISTS (d)were significantly predictive of marital satisfaction. Positiveregression coefficients for ISTS indicate that higher ISTS is associatedwith higher marital satisfaction. *P<0.05; ***P< 0.01.

The optimal number of events in the DMN differs across married couples with high-and low-level satisfaction, but this does not necessarily mean that the patternsof neural activity are unrelated. The coarser event structure in married coupleswith low-level satisfaction may still be present in married couples withlow-level satisfaction. It may be that some additional additive effect of highermarital satisfaction makes married couples segment these longer events morefinely. We thus investigated whether within HMM-denfied events, the ISTS inmarried individuals was associated with satisfaction. HMM event segmentationmodels were applied to DMN activity patterns in all married couples. This methodwas employed to ascertain whether a specific pair of TRs should be categorizedas “within-event” (i.e., if both TRs belong to the same event).Then, we examined within-HMM-event ISTS in each of the 200 ROIs for each marriedcouple (Section 2). Whenanalyzing the trajectory structures defined by HMM within events, we observed apattern of results similar to those in our main analyses. More specifically,compared with couples with low-level marital satisfaction, married couples withhigh-level marital satisfaction had greater within-event ISTS scores whenviewing movies (*t*= 2.96,*P*=0.006) (Fig. 6a). In fact, thefMRI stimuli consisted of 12 movie clips. Each clip has a theme, involvingmarried life, sex, children, food, objects, and architecture. Hence, wedelineated each natural segmentation of movie clips as an event and proceededwith an additional exploratory analysis, as outlined in theSection 2, investigating the intra-movie-clipsISTS for each married couple. Compared with couples with low-level maritalsatisfaction, married couples with high-level marital satisfaction also hadgreater within-movie-clips ISTS scores when viewing movies (*t*= 2.02,*P*= 0.05) (Fig. 6c). However, compared with thewithin-movie-clips ISTS (Fig.6d), the within-event ISTS in more extensive brain regionscontributed to marital satisfaction while viewing movies (Fig. 6b).

## 4 Discussion

Our prior research examined neural responses to naturalistic stimuli by focusing onfluctuations in overall response magnitude among married couples (Li et al., 2022). The findingsfrom the present study indicate that trajectories of spatially distributed responsepatterns offer another perspective on individual variations in response to thesestimuli. We found that married couples have more similar trajectories of MVPs thanrandomly selected pairs of couples when viewing movies. Higher ISTS between marriedcouples was associated with greater marital satisfaction. Furthermore, the ISTS ofMVPs within manually defined events was predictive of marital satisfaction above andbeyond the effects of the ISTS within inherent movie clips. These results suggestthat married couples with high levels of marital satisfaction may exhibitsignificant similarity in the evolution of their psychological states, particularlyin relation to social processing and endogenous attention. Our findings underscorethat the temporal trajectories of MVPs capture unique individual differences inneural responses, distinct from those captured by temporal fluctuations in regionalsignals. These results provide strong evidence for the link between couplesimilarity in terms of trajectories of psychological states and marital satisfactionand extend our understanding of couple similarity in mate selection and relationshipoutcome.

MVP trajectories effectively disregard the spatial layout of each subject’sdata, as individuals are compared based on the relationships between responsepatterns across time points rather than the direct response patterns themselves(Kriegeskorte & Bandettini,2007). The current approach, thus, considers spatial distribution ofresponse patterns and how these patterns change over time. In the present study, wefound that married couples exhibited greater similarities in the trajectories ofMVPs compared to random pairs. Multiple analyses confirmed that couples with highlevels of marital satisfaction had higher ISTS. Notably, this effect was specific todynamic, time-varying response patterns elicited by exposure to naturalisticstimuli. Our results also revealed a significant correlation between marriageduration and ISTS during the resting state, while no such significance was observedduring movie viewing. Previous research suggests that cultural contexts modulateresting-state neural similarity, providing a plausible explanation for thesefindings (Xu et al., 2023).Specifically, the higher resting-state similarity observed in married couples maystem from shared socio-cultural backgrounds and the cumulative effects of long-termsocial interactions. In contrast, the heightened neural similarity observed duringmovie viewing in couples with high marital satisfaction likely reflects matepreferences and shared emotional or cognitive resonance in response tomarital-themed stimuli. These findings collectively highlight the complex interplaybetween socio-cultural factors, long-term interactions, and individual preferencesin shaping neural synchrony within close relationships.

In addition to ISTS measures at the whole-brain level, our analyses identifiedspecific brain areas predictive of marital satisfaction. The most significanteffects were localized in regions of the DAN, which is associated with endogenouslydriven shifts in attention. DAN areas are also linked with episodic memoryretrieval, especially when attention is allocated to memories (Hutchinson et al., 2009). Giventhat all participants were exposed to the same stimuli, the way in which exogenouslydetermined attentional states fluctuate over time may not differ significantly.Therefore, our main findings may reflect how married couples allocate theirattention to specific stimuli or scenes based on their similar goals or memorieswhen viewing video clips. These results suggest that comparing perceivers based onchanges in the trajectory of MVPs over time, rather than solely on fluctuations inresponse amplitude, may offer a promising approach for capturing similaritiesbetween subjects (Haxby et al.,2014;Norman et al.,2006).

Next, we investigated neural event segmentation using a data-driven, computationalapproach in married couples with high and low levels of marital satisfaction whilethey viewed movies. We found synchronous processing of the movie and a reliableevent structure in both groups. However, married couples with low maritalsatisfaction exhibited coarse neural event structures across regions in the DAN andDMN. In contrast, compared to those with low marital satisfaction, movies evokedmore and shorter events in the DMN among couples with high marital satisfaction.This could be due to a tendency to segment events according to goals during encoding(Pérez et al.,2021).

Marital movie scenes are rich, engaging, and energetic, aligning well with the mentalprocesses involved in partners’ daily interactions, such as communication,sex, attitudes toward relatives, conflict resolution, financial disputes, and sharedvalues (Hasson et al., 2010;Li et al., 2022).Consequently, event boundaries detected in the brain may provide more“objective” insights into the similarity of couples’ mentalprocesses as they experience and react to the world around them. We then comparedthe neural response patterns of psychological states to those of correspondingevents across groups. We conducted an ISTS analysis, where ISTS were summarized byHMM-identified events in the DMN rather than by TRs. This finding contrasts withresults from similar analyses relating marital satisfaction to ISTS across the wholeexperiment. Compared to ISTS within inherent segmentation of movie clips, ISTSwithin HMM-identified events were more predictive of marital satisfaction. Thiscould be attributed to the association between marital satisfaction and similaritiesin spouses’ personality traits and previous marital experiences. Thisassociation may lead couples to perceive stimuli with similar goals, knowledge,memories, and expectations, thereby influencing which aspects of the stimuli aredeemed relevant or interesting at any given time (Hyon et al., 2020).

Notably, the DMN is a functionally heterogeneous, large-scale brain network involvedin a range of internal mental processes, including internally directed attention,self-referential thought, and social cognition (Menon, 2023). It also plays a crucial role in predictingindividuals’ current and future mental states (Thornton et al., 2019). Innovative studies employingnaturalistic stimuli have demonstrated that DMN activity synchronizes when twoindividuals process a shared narrative, with this synchronization being particularlysensitive to social and communicative cues (Yeshurun et al., 2021). Our previous research alsosuggested that neural synchronization within the DMN is associated with maritalsatisfaction in couples (Li et al.,2022). In this context, inter-subject synchronization within the DMN mayreflect similar mental representations, such as shared values, beliefs, andcomparable perceptual “styles” of the world (Menon, 2023). Previous work hasproposed that these shared representations within the DMN may arise from dynamicsocial interactions between the self and others. Through reciprocal and interactiveexchanges, the brain responses of one individual can influence those of another, andvice versa, thereby creating a shared cognitive reality (Yeshurun et al., 2021). Formarried couples, high neural alignment within the DMN may thus result from ongoingsocial interaction and coordination during day-to-day activities, such ascommunication, sexual interactions, managing financial disputes, resolvingconflicts, and discussing shared values and attitudes toward relatives. Theseinteractions foster continuous synchronization of mental states over time. Thecurrent findings build upon and extend these observations, suggesting that marriedcouples not only share neural synchrony but also exhibit temporal similarities intheir psychological states.

## 5 Conclusion

In summary, by analyzing the trajectory of distributed response patterns within brainregions, rather than considering only the overall response magnitude, a wealth ofinformation about mental states would be detected. We investigated the associationbetween ISTS to naturalistic audiovisual stimuli and marital satisfaction in marriedcouples. Compared to randomly selected pairs of couples, married couples showedsignificantly higher levels of ISTS during viewing of movies, and ISTS in DMN/DANbetween married couples predicted higher levels of marital satisfaction. Therefore,married couples may undergo a comparable ebb and flow of internally driven mentalstates throughout naturalistic stimulation. This phenomenon could stem fromsimilarities in individuals’ expectations, interests, and predispositionsrelated to marital life, which subsequently shape their allocation of attention tovarious elements of external stimuli and their internal values, beliefs, andmemories during the viewing of naturalistic stimuli.

However, we cannot draw conclusions from the current data on whether neuralsimilarity is a cause or consequence of successful marriage. Thus, longitudinalstudies should be adopted in the future to measure the important role of couplesimilarity in the neural response in mate selection and relationship maintenance andoutcomes. Moreover, although the naturalistic neuroimaging paradigm of this studyhas many advantages, a more detailed understanding of the cognitive and emotionalprocesses underlying these effects may require additional future studies involvingbehavioral measurements and more constrained experimental paradigms.

## Data Availability

Anonymized fMRI, demographic and behavioral data, and code used for analyses havebeen deposited in a Zenodo under (https://zenodo.org/records/11377487).
